# Nuclear Envelope Permeability Barrier as a Fast‐Response Intracellular Mechanostat

**DOI:** 10.1002/advs.201900709

**Published:** 2019-08-29

**Authors:** Victor Shahin, Ivan U. Kouzel, Gonzalo Rosso, Ivan Liashkovich

**Affiliations:** ^1^ Institute of Physiology II University of Münster Robert‐Koch‐Str. 27b 48149 Münster Germany; ^2^ Sars International Centre for Marine Molecular Biology University of Bergen Thormøhlensgt. 55 N‐5006 Bergen Norway; ^3^ Biotechnology Center Technical University Dresden Tatzberg 47/49 01307 Dresden Germany

**Keywords:** atomic force microscopy, mechanobiology, nuclear envelope

## Abstract

The nuclear envelope is an undisputed component of the intracellular mechanotransduction cascades which collect, process, and respond to mechanical stimuli from the environment. At the same time, the nuclear envelope performs the function of a selective barrier between the nuclear and cytoplasmic compartments. Although the mechanosensing and the barrier functions of the nuclear envelope have both been subjects of intense research, a possible reciprocal relationship between them is only beginning to emerge. In this report, the role of the nucleocytoplasmic permeability barrier is evaluated in nuclear mechanics. Using a combination of atomic force and confocal microscopy, the functional state of the nucleocytoplasmic permeability barrier and the nuclear mechanics is monitored. By modulating the stringency of the barrier and simulating the active transport imbalance across the nuclear envelope, the decisive impact of these parameters on nuclear mechanics is demonstrated. It is concluded that the nucleocytoplasmic barrier is the second essential component of the intracellular mechanostat function performed by the nuclear envelope.

## Graphical abstract

**A role of nucleocytoplasmic permeability barrier in nuclear mechanics** is demonstrated by simultaneous confocal and atomic force microscopy. A compromised barrier results in a 2.4‐fold reduction of the nuclear elastic modulus and a diminished ability to withstand dynamic mechanical loads. Proposed ultrafiltration model of nuclear mechanics integrates the barrier function of the nuclear envelope with its role as an intracellular mechanostat.

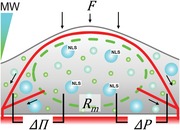

## 1 Introduction

The primary function of the nuclear envelope and the nuclear pore complexes (NPCs) is to transport a subset of intracellular molecules into and out of the nucleus while being a stringent barrier for the others.^1^, ^2^, ^3^, ^4^ Over the past two decades, it has become apparent that the nuclear envelope also plays a pivotal role in sensing, processing, and responding to the mechanical stimuli from the environment.^5^, ^6^ The importance of this function is underscored by the severity and the multitude of clinical manifestations associated with the mutated mechanical components of the nuclear envelope.^7^, ^8^, ^9^ Although a possible impact of the transport/barrier function of the nuclear envelope on nuclear mechanics has been suggested,^10^ the experimental studies have unduly disregarded this possibility. The reasoning behind including the transport function into the mechanical model of the nucleus is based on a functional similarity between the nucleus and an artificial ultrafiltration system. Such an artificial system is comprised of two compartments separated by a porous membrane with a given molecular weight cut‐off value. Once one of the compartments becomes pressurized, the gradient of pressure across the membrane drives the mass transport in a predetermined direction

(1) $$J\;=\frac{\Delta P-\Delta \Pi }{R_{m}}$$

The magnitude of this flux (*J*) depends largely on the magnitude of the pressure gradient (Δ*P*) and the resistance of the filter membrane (*R*_m_). As the volume of the pressurized compartment drops, the concentration of molecules above the permeability cut‐off increases creating a gradient of colloid‐osmotic pressure (ΔΠ) counteracting the filtration pressure as described by Wijmans et al.^11^ Similar to the artificial system, nuclear envelope separates intranuclear and cytoplasmic compartments from each other providing a stringent barrier for molecules above its permeability cut‐off.^12^ The nuclear compartment is pressurized by the cytoskeletal and environmental forces.^13^ The ability of the NPCs to actively transport molecules above the filter cut‐off and active regulation of the gradient of colloid‐osmotic pressure (ΔΠ) across the nuclear envelope distinguishes it from its artificial counterpart. This colloid‐osmotic mismatch across the nuclear envelope may have a significant impact on the nuclear rigidity. On the other hand, the number and the physiological state^14^, ^15^, ^16^, ^17^ of the NPCs can have an impact on the membrane resistance (*R*_m_) defining the rate of mechanical equilibration in response to external load. We hypothesize that if the nucleus can be modeled as a mechanically actuated ultrafiltration system, then its stress–strain response will depend not only on the nuclear structural components represented by the lamin network and chromatin.^5^, ^18^, ^19^ Additionally, the overall mechanics of the nucleus will also depend on the functional state of the NPCs.

In this report, we test this hypothesis by assessing the contribution of the transport/barrier function of the nuclear envelope toward determining the global mechanics of the nucleus. We: 1) investigate the impact of the unbalanced distribution of molecules above the barrier cut‐off (ΔΠ) across the nuclear envelope on nuclear mechanics; 2) modulate the stringency of the barrier (*R*_m_) and evaluate the associated changes of nuclear mechanics; 3) demonstrate that the mechanical response of the nucleus to dynamic loads (Δ*P*) depends on the integrity of the barrier; 4) incorporate our findings into the current paradigm of nuclear mechanics and discuss the implications for basic cell biology and pathophysiology.

## 2 Results and Discussion

### 2.1 Experimental Strategy for Validation of the Nuclear Ultrafiltration Model

**Figure 1:**
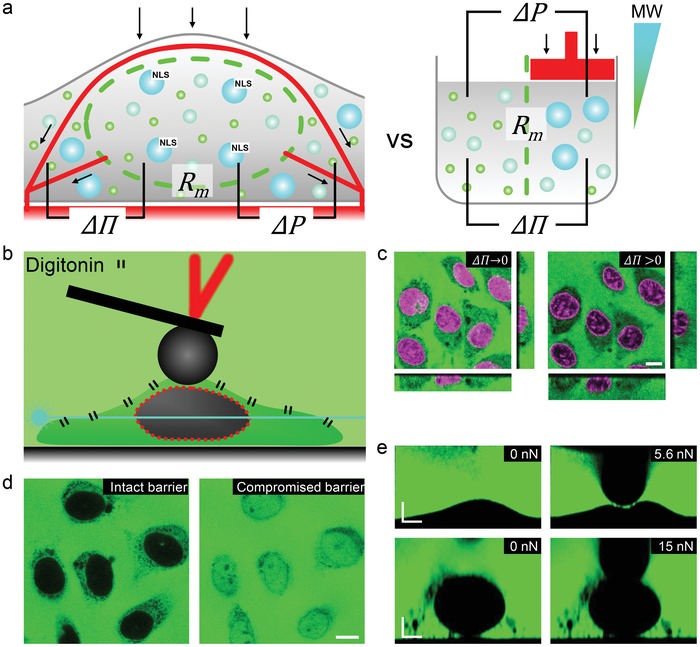
Ultrafiltration mechanical model of the nucleus and an experimental strategy for its validation. a) Schematic comparison of the mechanical operating parameters of an artificial ultrafiltration system (right) and a nucleus of a living cell (left). Similar to an artificial system, the nucleus may experience a trans‐nuclear envelope permeate flux under dynamic loading conditions (Δ*P*). The magnitude of this flux will depend on the physiological state and the number of the NPCs (*R*_m_). In addition, the effect of mechanical load may be counterbalanced by an increasing ΔΠ as the fluid escapes the nucleus while the large macromolecules stay trapped inside. b) Experimental setup includes digitonin‐permeabilized endothelial cells which can be simultaneously imaged with a confocal laser scanning microscopy and mechanically probed with AFM. c–e) Modulation of the operating parameters of the nuclear ultrafiltration system. c) Variation of the colloid‐osmotic content of the medium provides control of ΔΠ. d) Paired pharmacologic modulation of the NPC permeability barrier integrity allows modulation of *R*_m_. e) Application of mechanical forces by AFM is used as a strategy to control Δ*P*. Scale bars: c,d) 10 µm; e) 5 µm.

Modeling the operation of the nuclear envelope according to the principles of an ultrafiltration system (**Figure** **1**a) is based on understanding several key parameters. The most prominent ones are the macromolecular concentration asymmetry across the nuclear envelope together with the physiologically regulated stringency of the NPC cut‐off value. By acting together they would determine the gradient of the colloid‐osmotic pressure across the nuclear envelope and thus the mechanical response of the nucleus to external load

(2) $$\Delta P=\Delta \Pi +JR_{m}$$

To assess the validity of such a model of the nucleus, we need to be able to alter the putative operating parameters of the system (ΔΠ and *R*_m_) while monitoring the mechanical response of the nucleus. Altering and measuring these parameters in the living cells presents a considerable challenge. The balance of macromolecular distribution across the nuclear envelope is affected by a multitude of factors including the transport activity across the nuclear envelope, differing rates of macromolecular synthesis, and contractile activity of cytoskeleton. Although we have a solid understanding of the transport processes across the nuclear envelope, the net balance of the macromolecular distribution between the cytoplasm and the nucleus is poorly quantified. Cytoskeletal activity exerts external mechanical force on the nucleus potentially resulting in dynamic shift of the ΔΠ across the nuclear envelope. Finally, the requirement for a reliable approach toward modulation of the stringency of the NPC permeability barrier with a quantifiable real time read‐out within the context of a living cell adds yet another layer of complexity.

**Figure 2:**
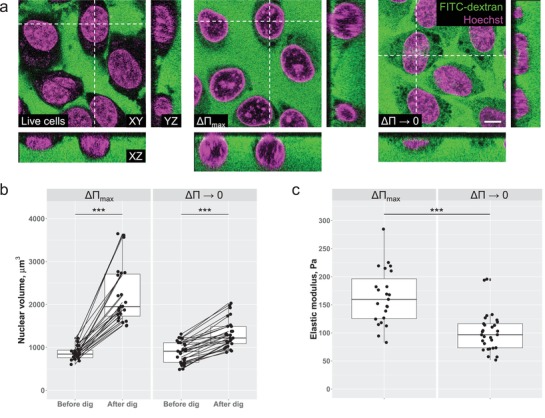
Colloid‐osmotic mismatch across the nuclear envelope affects the nuclear elastic modulus. a) Nuclear geometry of living cells (left) as compared to the geometry of the nuclei without (middle) and with (right) colloid‐osmotic compensation. The white dashed lines in XY images represent the location of XZ and YZ optical sections. Scale bar = 10 µm. b) Quantitative assessment of the nuclear volume changes at maximal (ΔΠ_max_) versus minimal (ΔΠ → 0) nucleocytoplasmic colloid‐osmotic mismatch. c) The effect of the magnitude of the colloid‐osmotic mismatch on the elastic modulus of the nuclei. *N* = 4, *n* = 24 for ΔΠ_max_, and *n* = 29 for ΔΠ → 0 for (b) and (c).

To reduce the complexity and to attain the necessary level of control required for accurate assessment of the impact the nuclear envelope permeability barrier may have on nuclear mechanics, we use digitonin‐permeabilized endothelial cells^20^, ^21^ as a biological model (**Figure** **2**b). Here, a mild selective disruption of the plasma membrane is achieved by a short (5 min) exposure to a digitonin solution in an aqueous buffer closely mimicking the intracellular ionic composition. Permeabilization efficiency can be monitored in the real time by confocal microscopy ensuring minimal exposure of the cells to digitonin and reducing potential unspecific activity. Once the plasma membrane is permeabilized, digitonin is washed out together with the chemical energy and cytosolic factors. At the same time, intranuclear macromolecules are retained within the nucleus due to their inability to cross the fully functional nucleocytoplasmic permeability barrier. This has several important consequences for accurate assessment of nuclear mechanics. The dissipation of chemical energy inactivates cellular processes which may have an impact on the studied system. Cytoskeletal, transport, and synthetic activities are fully disabled. As a result, the mechanical properties of the nucleus no longer depend on dynamic processes such as cytoskeletal activity or a nucleocytoplasmic imbalance of synthetic or transport activities while the material properties of the structural components, i.e., lamin network and chromatin are kept constant throughout the experiment. The impact of cell‐to‐cell variability in nuclear mechanics is further reduced by the paired mechanical probing of the same set of cells before and after respective treatments. Most importantly, digitonin permeabilization provides a direct unobstructed access to the nuclear envelope. This enables accurate assessment of the state of the nucleocytoplasmic permeability barrier by adding a fluorescent tracer to the medium (Figure 2d). It also grants a possibility to keep the ionic conditions constant throughout the experiment eliminating the possibility of the chromatin condensation contributing to the observed mechanical effects. Critically, digitonin permeabilization allows to carefully modulate the barrier cut‐off value (*R*_m_) pharmacologically^2^, ^3^ and the colloid‐osmotic pressure gradient (ΔΠ) by adjusting the macromolecular content of the medium. Taken together, our biological model enables exquisite control and quantification of the physiological state of the nucleocytoplasmic permeability barrier while ensuring the constant environment for the classical determinants of nuclear mechanics such as nuclear lamina and chromatin.

The changes in nuclear mechanics associated with the alteration of the nucleocytoplasmic barrier function are assessed by an atomic force microscope (AFM) coupled to a confocal laser scanning microscope. This synergistic combination of instruments^22^ allows gauging the impact of the nuclear envelope functional state on the overall mechanics of the nucleus (Figure 1e).

Confocal imaging performed concomitantly with the AFM probing supplies several crucial parameters. First, it provides the feedback on the functional state of the nuclear envelope permeability barrier. Second, it enables precise positioning of the AFM tip over the probed nucleus (Figure S1, Supporting Information). Critically, it allows accurately determining the exact geometry of the probed nuclei required for the analytical model we use here for calculating the elastic moduli of the probed nuclei^23^ (see the Experimental Section for the mechanical quantification). On the other hand, using AFM in conjunction with confocal microscopy allows us to correct for the previously described *Z*‐axis scaling aberrations^24^ when imaging in a medium with an optical density differing from that of the immersion medium. Taken together, the combination of the biological model and instrumentation described above allows us to systematically alter the operating parameters relevant for the assessment of the validity of the ultrafiltration model of nuclear mechanics. At the same time, possible interference from other factors known to affect nuclear mechanics is largely eliminated.

### 2.2 Nuclear Mechanics Depends on the Magnitude of Colloid‐Osmotic Pressure Gradient across the Nuclear Envelope

The critical difference between a nucleus and an artificial ultrafiltration system is that the active transport process, being the primary function of the NPC, is able to shift the balance of the colloid‐osmotic pressure (ΔΠ). This in turn may affect the overall pressure across the nuclear envelope and thus the nuclear mechanics as a function of the extent of nuclear deformation under external mechanical load. To simulate the nucleocytoplasmic transport asymmetries and their impact on nuclear mechanics, we assess the mechanical properties of the nucleus when ΔΠ approaches its maximal value (ΔΠ_max_) and compare it to the situation where ΔΠ → 0 (Figure 2).

The extent of ΔΠ is approximated as a function of the change of nuclear geometry upon digitonin permeabilization of the plasma membrane in presence of varying concentrations of unlabeled 70 kDa dextran and compared to the nuclear height in the living cells (Figure S2, Supporting Information). Titration of the nuclear height with varying concentrations of 70 kDa dextran reveals that the minimal change in nuclear geometry and volume occurs at 3% dextran which we use as a value of ΔΠ → 0. The ΔΠ_max_ is achieved by supplementing the permeabilized cells with a buffer devoid of dextran. The AFM probing of the nuclei at ΔΠ → 0 and ΔΠ_max_ reveals that the nuclei facing a strong mismatch of the colloid content across the nuclear envelope exhibit significantly higher values of the elastic modulus compared to the nuclei at ΔΠ → 0 (Figure 2c). These results show that at equilibrium conditions when the flux across the nuclear envelope is negligibly small, the mechanical properties of the nucleus indeed depend on the extent of the colloid‐osmotic mismatch between the intra and extranuclear compartments.

To unify our finding with the current paradigm of nuclear mechanics, we need to consider what happens with the major load‐bearing components of the nucleus and the dynamic stresses it experiences within a living cell upon digitonin permeabilization and subsequent alteration of the colloid content of the surrounding medium. Currently accepted view on nuclear mechanics states that the mechanical properties of the nucleus are determined by a dynamic interplay between the intrinsic material properties of the lamin network^5^ and the underlying layer of chromatin^19^ on one hand and the mechanical stresses exerted on these load‐bearing structures by cytoskeletal contractility.^13^ Lamins have been shown to unfold under mechanical stress. However, long‐term adaptation to excessive stress requires upregulation of lamin A/C expression.^25^ This type of adaptation cannot occur in our model system due to a short duration of the experiment and the lack of biochemical factors required to drive the increased expression. The same holds true for the dynamic cytoskeletal stress. Cytoskeletal dynamics is halted upon digitonin treatment due to the removal of chemical energy required for active actomyosin contractility. Chromatin is the only structural component which undergoes visible structural alteration as a result of plasma membrane permeabilization with digitonin. Chromatin staining performed during our experiments reveals that it displays a more condensed uniform structure similar to the in vivo situation at ΔΠ → 0 whereas at ΔΠ_max_ the chromatin density visibly decreases due to the fluid influx driven by the colloid‐osmotic mismatch across the nuclear envelope. However, the associated changes in nuclear mechanics are the opposite of what has been reported previously for the cells treated with chromatin decondensation agents^18^, ^19^ indicating the primary role of the colloid‐osmotic mismatch in nuclear stiffening. The fact that the ionic environment is kept constant while the only varied parameter is the colloid content of the medium makes it unlikely that the chromatin restructuring underlies the observed changes of nuclear mechanics. Based on the argument that the digitonin permeabilization allows us to vary only one parameter (ΔΠ) while keeping the others constant, we conclude that the nonuniform macromolecular distribution across the nuclear envelope affects the overall mechanics of the nucleus. This impact of the colloid‐osmotic mismatch across the nuclear envelope on nuclear mechanics has been observed in a simplified model system raising questions on the validity of our findings in an in vivo situation. Although we use an extreme level of colloid‐osmotic mismatch as one of our experimental conditions, it is not difficult to envision a situation within a living cell when the nucleocytoplasmic transport balance is shifted in favor of import. This could lead to an increase in the intranuclear macromolecule content and thus result in a steeper gradient of the colloid‐osmotic pressure across the nuclear envelope. To our knowledge, the contribution of this kind of mechanical stress experienced by the nucleus has not been reported previously let alone incorporated into the current models of nuclear mechanics. Therefore, we cannot refrain from speculating on the implications of our finding for cell physiology as well as pathological situations associated with dysfunctional mechanobiology of the nuclei. We would like to propose that the mechanical stability of the nucleus under physiological conditions may depend not only on the material properties of its structural components responding to external mechanical stresses. A possibility needs to be considered that these structural components can be subjected to additional mechanical stress which arises from an acute imbalance in nucleocytoplasmic transport or macromolecular synthesis. This possibility hints at a plausible albeit highly speculative explanation of why laminopathies affect not only mechanically challenged tissues but also soft adipose tissue.^26^ The already low nuclear envelope mechanical limit of adipose tissue cells expressing low levels of lamin A/C may be compromised even further due to mutations in this protein. Consequently, these cells are no longer able to sustain physiological levels of nucleocytoplasmic transport when the colloid‐osmotic pressure within the nucleus exceeds the mechanical limit of their weakened nuclear envelope. Nevertheless, further experiments are needed to establish the validity of this hypothesis.

### 2.3 Integrity of the Nucleocytoplasmic Permeability Barrier Is Required for Maintaining the Mechanical Properties of the Nucleus

**Figure 3:**
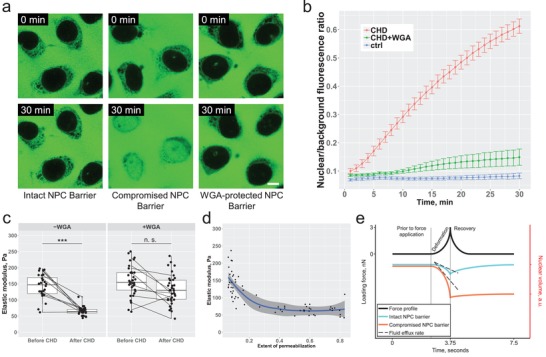
Integrity of the NPC permeability barrier determines the elastic modulus of the nucleus. a) Pharmacological modulation of the NPC permeability barrier with *trans*‐1,2‐cyclohexanediol. The barrier is strongly compromised after 30 min of exposure to CHD (middle). If the barrier is protected by pretreatment with WGA, the influx of dextran in presence of CHD is reduced to the control levels of untreated nuclei. b) A statistical summary of the dextran influx kinetics into the WGA‐protected versus unprotected nuclei treated with CHD as compared to the intact nuclei (*N* = 4 for each condition, *n* = 28 for CHD‐treated unprotected nuclei, *n* = 36 for CHD‐treated WGA‐protected nuclei, and *n* = 17 for control nuclei). c) Treatment with CHD results in a nearly twofold reduction of the nuclear elastic modulus (left) unless the nuclei are protected by WGA (right) (*N* = 4 for each condition, *n* = 28 for CHD‐treated unprotected nuclei, and *n* = 36 for CHD‐treated WGA‐protected nuclei). d) Dependence of the nuclear elastic modulus on the extent of the nucleocytoplasmic barrier permeabilization, *n* = 64. e) The extent of nuclear deformation in response to a dynamic short‐term mechanical load depends on the functional state of the nucleocytoplasmic permeability barrier. Higher rate of fluid efflux through a compromised barrier results in a higher deformation of the nucleus.

The next parameter which strongly impacts the performance of an artificial ultrafiltration system is the membrane resistance (*R*_m_). In the nuclear envelope, this parameter is determined by the absolute number of the NPCs and their functional state. A critical distinction of the NPC from an artificial pore is its flexible modular composition,^27^, ^28^, ^29^ which can be subjected to alterations through several signaling pathways.^16^ As a result, a cell gains an ability to modulate the stringency of the NPC permeability barrier. However, the effect of such modulation on the mechanics of the nucleus has not been investigated to date. We alter the stringency of the NPC permeability barrier pharmacologically and assess the resulting impact on nuclear mechanics. We employ *trans*‐1,2‐cyclohexanediol (CHD) which relaxes the stringency of the barrier.^2^, ^3^ To exclude the possibility of nonspecific CHD effects, we also assess the mechanics of the nuclei which are protected from CHD by preincubation with wheat germ agglutinin (WGA) (**Figure** **3**; Figure S3, Supporting Information).

Time‐lapse confocal microscopy confirms that application of CHD compromises the integrity of the nucleocytoplasmic barrier while the nuclei protected by WGA remain largely unaffected by CHD. Mechanical probing reveals that the integrity of the barrier is critical for maintaining the rigidity of the nucleus (Figure 3c) and that a slight increase in the extent of NPC permeabilization leads to a significant reduction of the nuclear resilience to mechanical loads. Before we proceed with interpretation of the obtained results, several important technical aspects of the experiment need to be considered. The starting biomaterial used for the experiment is the digitonin‐permeabilized cells identical to those characterized under the ΔΠ_max_ condition in the previous section. In these experimental settings, the nuclei are characterized by an increased volume and a loosening of chromatin organization. Neither the chromatin organization nor the nuclear geometry seems to change upon addition and incubation in CHD (Figure S4, Supporting Information). This, together with two additional controls strongly supports the notion of the observed mechanical alterations being dependent on the integrity of the nucleocytoplasmic permeability barrier. First, protection of the NPCs by WGA abolishes the effect of CHD. Second, disruption of the lipid component of the nuclear envelope by 0.1% Triton X‐100 even in presence of WGA results in a significant reduction of nuclear rigidity (Figure S3, Supporting Information). To facilitate the interpretation of our observations within the framework of the ultrafiltration model of nuclear mechanics, we start by assuming a constant level of the colloid‐osmotic pressure across the nuclear envelope regardless of the state of the permeability barrier. In this case, our model (Equation (2)) is reduced to a simple representation of the Ohm's law. Under such conditions, the model predicts that if an identical mechanical load (Δ*P*) is applied, the permeate flux across the nuclear envelope of the nuclei with a compromised barrier and reduced *R*_m_ will be higher. The higher permeate flux across such nuclear envelope will then translate into a faster reduction of the nuclear volume and a correspondingly higher deformation (Figure 3e, time period between 0 and 3.75 s). Although the assumption of a constant level of the colloid‐osmotic pressure across the nuclear envelope is helpful for interpreting the effect of the compromised permeability barrier on the nuclear mechanics, we would like to stress that this assumption is not entirely valid in our model system. Previous work demonstrated that the application of CHD results in an efflux of macromolecules from the nuclei^3^ thus lowering the gradient of the colloid‐osmotic pressure. For this reason, we suspect that a compromised nucleocytoplasmic permeability barrier may have a twofold effect on the mechanics of the nucleus. First, a compromised permeability barrier provides less resistance to a permeate flux induced by external mechanical force. Second, a “leaky” barrier is no longer able to generate physiological levels of the colloid‐osmotic pressure across the nuclear envelope rendering the nucleus more deformable under external mechanical load as demonstrated in the previous section. Although the impact of colloid‐osmotic mismatch on nuclear shape has been demonstrated previously,^30^ this is to our knowledge the first demonstration of a direct dependence of the mechanical state of the nucleus on the nucleocytoplasmic permeability barrier integrity. A full extent of the fluctuations of the NPC permeability barrier stringency in living cells remains poorly characterized. However, based on several previous studies,^31^ it is safe to assume that the NPC cut‐off value does not remain constant but may be a subject to a tight regulation providing another mechanism for fine‐tuning nuclear mechanics.

### 2.4 Compromised Nucleocytoplasmic Barrier Reduces the Ability of the Nucleus to Buffer Short‐Term Dynamic Mechanical Loads

**Figure 4:**
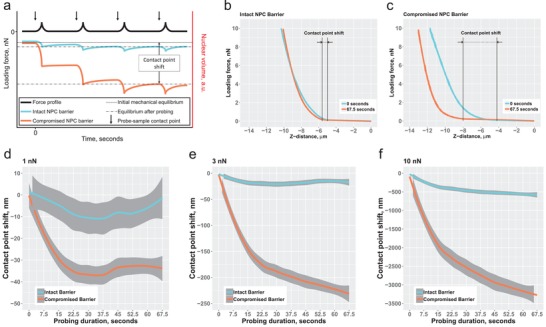
Nuclear deformation in response to a dynamic periodic mechanical load depends on the integrity of the NPC permeability barrier. a) Schematics of a hypothetical nuclear response to rapidly repeated mechanical loading as predicted by the ultrafiltration model. Under identical load, the nuclei with a compromised permeability barrier are expected to sustain larger deformations due to a diminished ability to recover from a previous cycle of mechanical loading. b,c) Nuclear response to dynamic mechanical loading quantified by measuring the shift of the probe‐sample contact position between the first and the tenth probing cycle. The shift is significantly increased in cells where the integrity of the nucleocytoplasmic barrier is b) compromised compared to c) cells with the intact barrier function. d–f) Nuclei with a compromised permeability barrier are unable to withstand progressively increasing loading forces as efficiently as the intact nuclei. This manifests as a strong shift of the probe‐sample contact point over the course of ten consecutive probing cycles normalized against the probe‐sample contact point position in cycle 1. *N* = 4, *n* = 28 for 1 and 3 nN, and *n* = 17 for 10 nN.

Nuclear envelope has been demonstrated to participate in buffering long‐term mechanical loads by upregulating the synthesis lamin A/C.^25^ Mechanisms responsible for dealing with the short‐term mechanical fluctuations involving nuclear envelope are less well defined. Ultrafiltration model of nuclear mechanics suggest a hypothetical mechanism of nuclear adaptation to short‐term high frequency mechanical loads. What would be the operating principle of such a fast response mechanostat? Based on the notion that mechanical load results in a fluid efflux from the nucleus, we can expect that the macromolecules within the nucleus confined to a smaller volume are now exerting a higher colloid‐osmotic pressure on the nuclear envelope. This could provide increasing mechanical resistance to further deformation by the external force. As soon as the mechanical stimulus is released, the increased colloid‐osmotic pressure together with the elastic recoil of the nuclear structural elements, i.e., lamin network and chromatin will contribute to the elastic recoil of the nucleus to the original volume and shape (Figure 3e, recovery section of the nuclear volume curves). Once the permeability barrier is compromised, the larger volume change induced by the external mechanical load together with a lower gradient of colloid‐osmotic pressure across the nuclear envelope may result in a slower rate of recovery from the deformation. This may have a significant impact when the nucleus is exposed to a sequence of brief mechanical stimuli following each other in a rapid succession (**Figure** **4**a). Slower recovery rate of the nuclei with a compromised permeability barrier will no longer be able to sustain the repeated mechanical loading resulting in a comparatively large deformation. Eventually, the nucleus may still be able to equilibrate with the external force once the nuclear volume has been reduced to a point where the colloid‐osmotic pressure matches the external load. The nuclei with an intact permeability barrier on the other hand should be able to buffer the same load much quicker without suffering excessive deformations. To test this hypothesis, we subjected the nuclei of digitonin‐permeabilized cells to ten consecutive probing cycles applied at an interval of 7.5 s. The key aspect of the experiment is the fact that the starting position of the AFM probe above the nucleus remains constant. This allows us to follow the dynamics of the nuclear recovery between two consecutive cycles of deformation by monitoring the distance the AFM probe covers between its initial position and the point where the contact with the cell is made. By comparing this distance between respective probing cycles, a contact point shift is calculated as illustrated in Figure 4b,c.

Direct overlay of a force‐vs‐distance curve obtained during the first probing cycle with a curve obtained during the tenth iteration of probing reveals that at the loading force of 10 nN, the nuclei with an intact permeability barrier respond very differently once the barrier integrity has been pharmacologically compromised. Paired probing of the same set of nuclei ensures that the observed phenomenon results from the effects induced by CHD. The nuclei with an intact barrier demonstrate a shift in contact point of 0.5 µm at the loading force of 10 nN over the course of 67.5 s. Relaxing the stringency of the NPC permeability barrier with CHD results in a sixfold increase of the contact point shift signifying a dramatic reduction of the ability of the nuclei to withstand high frequency external mechanical loads. To evaluate the temporal dynamics of the nuclear response to mechanical loads of three different values, we plot the contact point shift occurring over the course of ten consecutive probing cycles (Figure 4d–f). The results reveal that the nuclei with an intact barrier are more resilient to mechanical loads at each time point. The intact nuclei reach a state of mechanical equilibrium within the probing period as illustrated by a plateau phase. On the other hand, pharmacological relaxation of the barrier stringency has a profound effect on the ability of the nuclei to reach a mechanical equilibrium. Only at the loading force of 1 nN are the nuclei treated with CHD able to reach an equilibrium. Once the force is increased beyond 1 nN (3 and 10 nN), the nuclei are no longer able to withstand such mechanical loads as is evident from a progressively increasing shift of the probe‐sample contact point. The observed mechanical response of the nuclei with a compromised NPC permeability barrier underscores the importance of the barrier integrity not only for determining the overall mechanics of the nuclei but also for the response toward dynamic mechanical loads. We suspect that this ability to buffer high frequency mechanical stimuli which critically depends on the integrity of the nucleocytoplasmic permeability barrier serves as a mechanistic basis for a fast‐response mechanostat function of the nucleus.

### 2.5 Integration of the Nuclear Envelope Transport and Barrier Functions into the Model of Nuclear Mechanics

**Figure 5:**
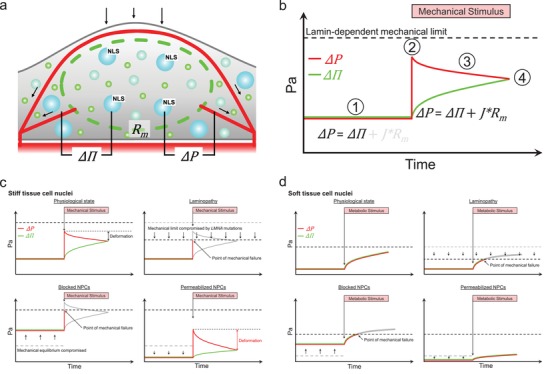
Ultrafiltration mechanical model of the nucleus. a) Schematics of the proposed model. Mechanics of the nucleus is suggested to be defined as a dynamic equilibrium between external forces (Δ*P*) and the gradient of colloid‐osmotic pressure (ΔΠ) established by the activity of NPCs. In addition, physiological state and the number of NPCs define the overall membrane resistance (*R*_m_). b) A hypothetical dynamic behavior of the nuclear ultrafiltration system. At equilibrium (1), permeate flux across the nuclear envelope is negligible (grayed out) and the mechanics of the nucleus is defined by the gradient of colloid‐osmotic pressure across the nuclear envelope. At the onset of a mechanical stimulus (2), the mechanical pressure increases initiating permeate flux across the nuclear envelope (3) which continues until the force is equilibrated by the colloid‐osmotic pressure (4). The maximal force the system can cope with depends on the level of lamin A/C expression defined here as the “lamin‐dependent mechanical limit.” Proposed mechanisms of the nuclear “ultrafiltration” model function under physiological conditions and in pathologies associated with lamina or NPC defects in c) stiff mechanically challenged tissues and d) soft tissues.

The experimental results described in previous sections demonstrate that the transport/barrier function of the nuclear envelope affects nuclear mechanics. To our knowledge, this is the first time that the barrier function of the nuclear envelope has been directly tied to the way in which a nucleus responds to an external mechanical challenge. Further in‐depth investigation of this relationship is required to assess the full extent to which the barrier/transport functions are interconnected with the nuclear mechanobiology. Nevertheless, we would like to present a preliminary hypothetical model to illustrate the impact the functional state of the nucleocytoplasmic permeability barrier may have on nuclear mechanics. So far our discussion of the role of the nuclear envelope permeability barrier function in nuclear mechanics has largely disregarded the contribution of the established nuclear load‐bearing components, namely, the lamin network and the underlying chromatin. We believe that all these components can still be tied together within the framework of the ultrafiltration model of nuclear mechanics (**Figure** **5**a,b).

Nuclear envelope and the NPCs represent the filter membrane while external mechanical forces generate a gradient of pressure across it. The ability of this pressure to drive material transfer across the nuclear envelope has been demonstrated recently by Elosegui‐Artola et al.^32^ On the other hand, the active transport across the NPCs together with the nucleocytoplasmic asymmetries in macromolecular synthesis generate a gradient of colloid‐osmotic pressure counteracting external mechanical loads. An equilibrium between ΔΠ and Δ*P* defines the mechanical properties of the nucleus. However, the actual position of the equilibrium cannot be arbitrarily high due to a fragile structure of the nuclear envelope contrasting with an artificial ultrafiltration membrane system able to withstand very high pressure differences. In the case of the nucleus, the upper limit of such a mechanical equilibrium between the external mechanical load and the colloid‐osmotic pressure across the nuclear envelope will be defined by the level of lamin A/C expression. Forces beyond this limit will result in a mechanical rupture of the nuclear envelope.^33^ Under physiological conditions well below this limit, the functional state of the nuclecytoplasmic permeability barrier plays a prominent role in defining both the static elasticity of the nucleus and the mechanical response to high frequency intermittent mechanical loads. Under static loading conditions, the ability of the nucleus to withstand mechanical load will not only depend on the level of lamin A/C expression but will also be influenced by the function of the NPCs. Active transport processes mediated by NPCs determine the magnitude of colloid‐osmotic pressure gradient across the nuclear envelope counterbalancing the static external load. Under dynamic loading, the magnitude of the pressure‐driven flux across the nuclear envelope is a function of the number of NPCs and the stringency of their permeability barrier. Therefore, the physiological state of the NPC permeability barrier will determine the rate of mechanical equilibration serving as a tunable low‐pass mechanical filter. This way excessive deformation of the nuclei under intermittent mechanical load could be prevented. The ability of the nucleus with an intact permeability barrier to buffer intermittent mechanical loads provides the missing short‐term component of the intracellular mechanostat function of the nucleus.^25^, ^34^ Taken together, our proposed model predicts that the external forces acting on the nucleus can be counterbalanced by the colloid‐osmotic mismatch across the nuclear envelope generated by both the transport and the barrier function of the NPCs. Critically, those force cannot exceed the upper mechanical limit of the nuclear envelope imposed by the level of lamin A/C expression.

Although extensive additional research is needed to verify the validity of this model in vivo, we would like to speculate on its implications for pathophysiology of syndromes associated with dysfunctional mechanobiology of the nucleus (Figure 5c,d). An unexpected but potentially highly interesting consequence of modeling the nucleus as a mechanically actuated ultrafiltration system is that the nuclear envelope may have to deal with two types of mechanical stress. On one hand, the nucleus has to withstand the external mechanical loads including the cytoskeletal forces. On the other, the active transport across the NPC and synthetic activity within the nucleus may generate a colloid‐osmotic mismatch which could potentially stress the nuclear envelope as well. We suspect that the relative contribution of these two types of stresses varies among different tissues. The stiff mechanically challenged tissues dealing with the excessive external mechanical stress cope by expressing high levels of lamin A/C. On the other hand, soft tissues dealing with comparatively low metabolically induced fluctuations of colloid‐osmotic pressure are able to contain it by low levels of lamin expression. In this context the mechanical resilience of the lamin network determined by the level of lamin A/C expression governs the amount of stress a nucleus can take before it mechanically fails. The amount of stress is dictated not only by the external mechanical forces but also by the colloid‐osmotic mismatch across the nuclear envelope stemming from the transport activity of the NPCs.

Expanding the model toward pathological cases provides a plausible albeit highly speculative explanation of the pathophysiologic effects of mutations associated with both the integrity of the lamin network and the NPCs. While the detrimental effects of the lamin A/C mutations on tissues exposed to extensive mechanical stress have been ascribed to the inability of the nuclei to cope with elevated external mechanical stress levels, the effect of certain laminopathies on soft adipose tissue remain unresolved. We suggest that the bursts of transport and macromolecular synthesis should also be considered as processes capable of mechanically stressing the nuclear envelope through changes in colloid‐osmotic pressure. In this case, the compromised lamina of the soft tissue may no longer be able to withstand the physiological levels of stress generated by the NPC transport activity. Another crucial implication is that the altered functionality of the NPCs even at physiological levels of lamin A/C expression may also have a detrimental effect on nuclear mechanics. In this case, altered rates of transport across the nuclear envelope may create an imbalance in colloid‐osmotic pressure which could exceed the mechanical limits of the lamin network. Despite our thorough understanding of the mechanisms of nucleocytoplasmic transport, we still have no quantitative assessment of the resulting imbalance of macromolecular distribution across the nuclear envelope under either physiological or pathophysiological conditions. However, several recent publications describing pathological phenotypes associated with nucleoporin mutations causing cardiac and nephrotic syndromes^35^, ^36^, ^37^ or interference with the NPC functions by the Tau protein in Alzheimer's disease^15^ lend indirect support for this hypothesis. On the other hand, reduction of the stringency of the NPC permeability barrier as described for ageing cells^14^ may impact the ability of the nucleus to serve as a short‐term mechanostat. The resulting outcome may be dictated by an inability of the lamin network alone to withstand excessive mechanically induced deformation which may lead to genomic instability and further cellular deterioration.

Finally, modeling the metazoan cell nucleus as a thin pressurized shell offers a hypothetical explanation of a hitherto puzzling absence of lamin‐like structures in the nuclei of plants and fungi. These organisms rely on extracellular cell wall structures to serve as a pressure container for growth and protection of cellular interior. Relegation of this function of the pressurized container to the nucleus through forming a dense network of lamins has equipped metazoan cell with a greater degree of flexibility in interacting with and adapting to their environment.

## 3 Conclusions

In this report, we investigated the functional interplay between the mechanical and the transport/barrier function of the nuclear envelope using a combination of a confocal laser scanning and atomic force microscopy. Taking advantage of the ability to simultaneously assess both the mechanical state and the integrity of the nucleocytoplasmic permeability barrier, we were able to correlate these two central functions of the nuclear envelope. The obtained results demonstrate that when both mechanical and the transport/barrier functions of the nuclear envelope are considered within the same context, they appear to reciprocally affect each other. The observed influence of the nuclear envelope transport/barrier function on nuclear mechanics can be accounted for by modeling the nucleus as a mechanically actuated ultrafiltration system. Despite being exceedingly oversimplified, the model is able to make accurate predictions concerning the changes of nuclear mechanics under both static and dynamic mechanical loads. The ability of the nuclei with the intact permeability barrier to buffer short mechanical stresses represents the missing short‐term component of the intracellular mechanostat function of the nucleus. Considering the potential implications of the model, it may be able to shed new light of the pathophysiologic mechanisms associated not only with laminopathies but also with genetic defects of the nucleocytoplasmic transport functions. Moreover, the ultrafiltration model of metazoan nuclear mechanics suggests a hypothetical function‐driven mechanism explaining the nature of the evolutionary divergence of nuclear structure among different kingdoms of eukaryotic organisms.

## 4 Experimental Section

*Cell Culture*: Ea.hy 926 endothelial cells kindly provided by Edgell et al.^21^ (University of North Carolina, Chapel Hill, NC, USA) were cultured at 37 °C, 5% CO_2_, and 100% humidity in minimal essential medium containing 1% nonessential amino acids, 1% MEM vitamins (Invitrogen Corp., Karlsruhe, Germany), and 10% fetal calf serum (FCS, PAA Clone, Coelbe, Germany). For imaging, cells were cultured on glass bottom petri dishes (WillCo Wells B. V., Amsterdam, The Netherlands).

*Modulation of the Functional State of the Nuclei*: The unobstructed access to the nuclei required for the modulation of the functional state of the nuclear envelope permeability barrier was provided by specific plasma membrane permeabilization with digitonin (Merck, Darmstadt, Germany). Permeabilization was achieved by exposing the cells to a solution of digitonin (20 µg mL^−1^) in transport buffer (TB, 110 × 10^−3^ m K‐Acetate, 5 × 10^−3^ m Na‐Acetate, 2 × 10^−3^ m Mg‐Acetate, 1 × 10^−3^ m EGTA, and 20 × 10^−3^ m HEPES pH 7.3) for 5 min. Subsequently, the permeabilization buffer was replaced with digitonin‐free TB containing 200 µg mL^−1^ 70 kDa FITC‐dextran (Merck, Darmstadt, Germany) which was required for the subsequent confocal imaging. For simulating the nucleocytoplasmic colloid‐osmotic mismatch, the transport buffer was supplemented with progressively increasing amounts of unlabeled dextran (Merck, Darmstadt, Germany) to achieve the concentrations of 0%, 3%, 5%, and 10%. The transport buffer was also supplemented with 10 µg mL^−1^ Hoechst 33342 (Life Technologies GmbH, Darmstadt, Germany) to facilitate the accurate assessment of nuclear volume by confocal 3D imaging. The assessment of the nuclear morphology (Figure S2, Supporting Information) revealed that at the extranuclear colloid concentration of 3%, the nuclear geometry and volume were closely matching the situation in living cells whereas higher concentrations resulted in excessive nuclear flattening. Consequently, transport buffer containing 3% dextran was chosen to represent the condition where ΔΠ → 0 during AFM probing. Modulation of the NPC permeability barrier integrity was performed as described previously.^2^, ^3^ In brief, the cells permeabilized with digitonin as described above and probed with AFM were subjected to treatment with 4% *trans*‐1,2‐cyclohexanediol (Merck) for a period of 30 min. The influx of the dextran was assessed by time‐lapse confocal imaging at the rate of 1 frame min^−1^. The cells were probed by AFM immediately after nuclear envelope permeabilization. To rescue the nuclei from the effect of CHD, the cells were permeabilized with digitonin in presence of 200 µg mL^−1^ wheat germ agglutinin (Merck). Protected nuclei were subsequently probed with AFM. After mechanical probing, the buffer was replaced with CHD‐containing buffer and the treatment was carried out as detailed above for the cells with unprotected permeability barrier. To ensure that the effect of WGA is based on its permeability barrier protective activity and not due to WGA binding elsewhere, the barrier integrity of nuclei protected with Alexa‐647‐labeled WGA (Life Technologies) was disrupted by treatment with Triton X‐100 (Carl Roth GmbH, Karlsruhe, Germany) and the AFM probing was repeated (Figure S3, Supporting Information).

*Confocal Laser‐Scanning Microscopy Imaging and Data Analysis*: Leica SP8 confocal laser scanning microscope equipped with a hybrid detection system for photon counting and an HC PL Apo CS2 63× NA 1.4 oil immersion objective (Leica, Wetzlar, Germany) coupled to a NanoWizard 3 atomic force microscope (JPK, Berlin, Germany) was used to monitor the functional state of the cells and their nuclei and probe their mechanics. Several modes of imaging were employed to obtain the data pertinent for individual aspects of this report. Single scans through the mid‐plane of the nuclei and the AFM probe were used to ensure the correct probe‐sample positioning right before probing (Figure S1c–e, Supporting Information). This proved particularly important for probing the living cells prior to digitonin permeabilization due to significant cell mobility. Confocal 3D stacks were acquired to obtain accurate information on nuclear volume and the overall sample geometry. Time‐lapse confocal imaging was utilized to monitor the integrity of the nuclear envelope permeability barrier and to assess the kinetics of fluxes in nuclei with compromised permeability barrier. The imaging data was processed and analyzed with ImageJ 1.51h (National Institutes of Health, USA). The nuclear volume for the colloid‐osmotic mismatch experiments was obtained by calculating a sum of the nuclear area in each slice of the 3D stack and multiplied by a corrected *Z*‐distance between slices based on the refractive index mismatch.^24^ The validity and the accuracy of the *Z*‐correction factor was confirmed by imaging the AFM probe positioned at defined distances from the sample surface. The extent of nuclear permeabilization was calculated by normalizing the mean intranuclear fluorescence intensity against the background fluorescence intensity.

*AFM Probing and Data Analysis*: Mechanical probing was performed with the NanoWizard 3 AFM driven by “JPK NanoWizard Control” v5.0.134 software. Silicon no‐reflex cantilevers with a nominal spring constant of 0.03 N m^−1^ functionalized with a 10 µm polystyrene sphere (Novascan Technologies, Inc. Boone, IA, USA) were used for all AFM‐probing experiments. The lack of reflex coating proved to be essential for a stable noise‐free tip‐sample contact during confocal imaging. Deflection sensitivity was calibrated prior to each experiment and was found to be consistently uniform throughout the whole experimental series. The *Z*‐scan size was set to the maximum scanning capacity of the scanner (15 µm) to ensure accurate assessment of the probed cell height as described previously.^38^ Mechanical probing was performed at forces of 1, 3, and 10 nN for ten consecutive cycles for each cell at each experimental condition. The resulting force–distance curves were analyzed with the JPK Data Processing v. spm‐5.0.134 software. The values of deformation under each respective loading force and experimental condition along with the initial sample heights obtained from confocal 3D stacks were used for calculating the elastic moduli. The calculations were based on the model of a soft finite thickness material layer on a rigid substrate developed by Dimitriadis et al^23^

(3) $$F\;=\frac{4E\sqrt{R_{c}}}{3\left(1-\nu^{2}\right)}\;\cdot \delta^{\frac{3}{2}}\left[1+1.133\chi +1.283\chi^{2}+0.769\chi^{3}+0.0975\chi^{4}\right]$$

where *F* is force in Newton (N); *R*_c_ is the radius of the probing sphere in meter (m); *E* is the elastic modulus in Pascal (Pa); ν is the Poisson ratio of 0.5; δ is the indentation in meter (m); $\chi \;=\sqrt{\delta R_{c}}/h$; *h* is the initial height of the sample determined by confocal microscopy for each probed cell in meters (m).

*Statistical Analysis*: Data were analyzed and visualized using R version 3.4.0 (2017‐04‐21)^39^ and R package ggplot2.^40^ The significance of the nuclear volume changes with and without osmotic compensation as compared to intact cells (Figure 2b) were assessed with a dependent 2‐group Wilcoxon Signed Rank Test. The alteration of nuclear volume was significant under both uncompensated conditions (ΔΠ_max,_ *p*‐value = 2.384e^−7^) and partially colloid‐osmotically compensated by addition of 3% 70 kDa unlabeled dextran (ΔΠ → 0, *p*‐value = 4.768e^−7^). The elastic moduli of the nuclei under the two types of experimental conditions (Figure 2c) (uncompensated vs partially colloid‐osmotically compensated) were significantly different as revealed by an independent 2‐group Mann–Whitney *U* Test (*p*‐value = 2.007e^−6^). Dependent 2‐group Wilcoxon Signed Rank Test was applied to the probing data of the nuclei with WGA‐protected versus unprotected nuclear envelopes upon being exposed to CHD (Figure 3c). The results show that CHD caused a significant drop of nuclear elasticity of unprotected nuclei (*p* = 2.235e^−8^) whereas WGA‐protected nuclei showed no significant elasticity change upon addition of CHD (*p*‐value = 0.362). The graphs in Figures 3d and 4c–e represent the data plotted with locally estimated scatterplot smoothing (LOESS). The data in Figure 3b represents mean nuclear/background intensity ratio values ± standard error.

## Conflict of Interest

The authors declare no conflict of interest.

## Supporting information

Supplementary
