# Supplementary material for: Nuclear Envelope Permeability Barrier as a Fast‐Response Intracellular Mechanostat
Source: Adv Sci (Weinh). 2019 Aug 29;6(21):1900709. doi: 10.1002/advs.201900709 (PMC6839651; doi:10.1002/advs.201900709)
Supplement: Supplementary file 1 — Supplementary [file ADVS-6-1900709-s001.pdf]

Copyright WILEY-VCH Verlag GmbH & Co. KGaA, 69469 Weinheim, Germany, 2019.

Supporting Information

for *Adv. Sci.*, DOI: 10.1002/advs.201900709

Nuclear Envelope Permeability Barrier as a Fast-Response Intracellular Mechanostat

*Victor Shahin, Ivan U. Kouzel, Gonzalo Rosso, and Ivan Liashkovich**

Nuclear envelope permeability barrier as a fast-response intracellular mechanostat

*Victor Shahin[1], Ivan U. Kouzel[2], Gonzalo Rosso[3, 1], and Ivan Liashkovich[1]**

Affiliations:

[1] Institute of Physiology II, University of Münster, Robert-Koch-Str. 27b, 48149 Münster, Germany

[2] Sars International Centre for Marine Molecular Biology, University of Bergen, Norway

[3] Biotechnology Center, Technische Universität Dresden, Tatzberg 47/49, 01307 Dresden, Germany

* Correspondence to: liashkov@uni-muenster.de

ORCID 0000-0002-0025-6124

University of Münster,

Robert-Koch-Str. 27b,

48149 Münster, Germany

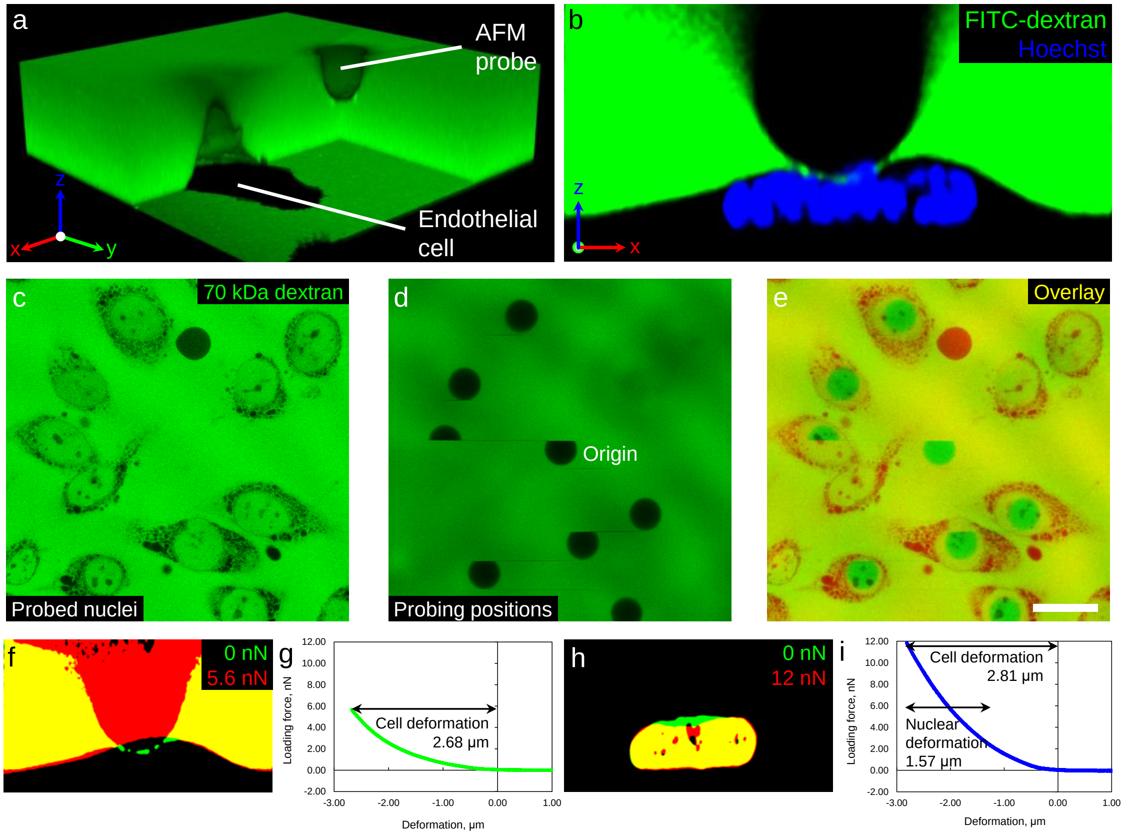

Figure S1. Combination of confocal and atomic force microscopy for probing nuclear mechanics. a) 3D volume rendering of a negatively stained endothelial cell and an AFM probe imaged by confocal microscopy. b) Optical readout of cell deformation under mechanical load applied by 10 µm AFM probe. Staining of the nucleus provides direct evaluation of the nuclear deformation. c-e) Confocal-assisted positioning of the AFM probe. Confocal scans at the cell focal plane c) and the AFM probe focal plane d) are overlaid e) to illustrate the precision of the AFM probe positioning above the probed nuclei. f) Computational overlay of binarized confocal images obtained at 0 nN and at 5.6 nN provide an optical readout of cell deformation. g) Cell deformation readout from the AFM force-deformation curve. h) Direct optical evaluation of nuclear deformation compared with i) total cell deformation.

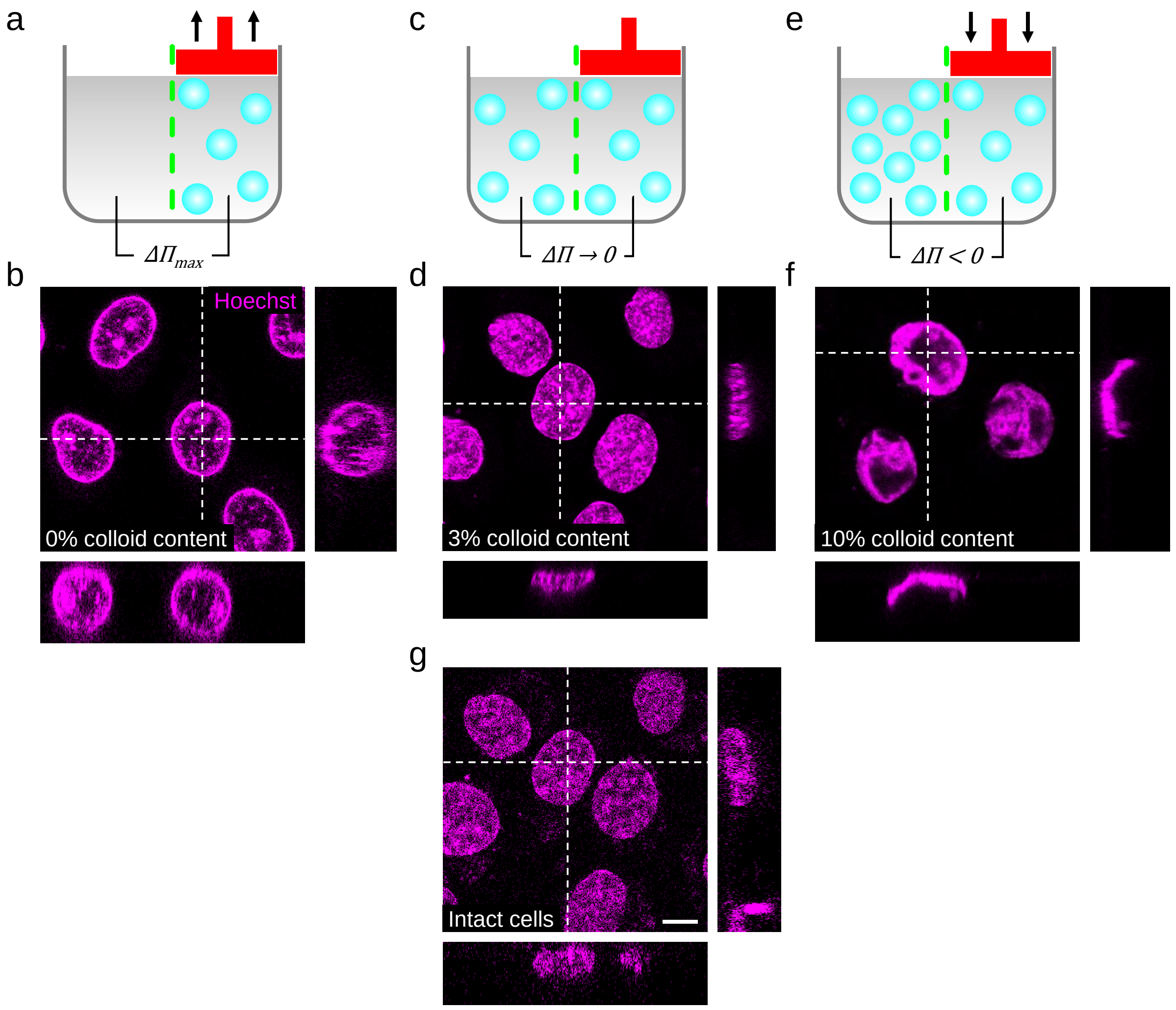

Figure S2. Modulation of the colloid-osmotic mismatch across the nuclear envelope. a) Maximal value of colloid-osmotic pressure is achieved by removing macromolecues from the environment. In this case colloid-osmotic pressure pushes against the “plunger”. b) Under similar conditions the nuclei exhibit increased volume due to colloid-osmotic extension of the nuclear envelope. c, d) When the colloid content is matched between the two compartments there is no pressure difference experienced by the membrane. In this situation the volume of the nuclei matches that of the nuclei of the intact cells (g). e, f) Excessive colloid content reverts the direction of the colloid-osmotic pressure experienced by the membrane. Scale bar = 10 µm. White hatched lines represent the positions of XZ and YZ optical sections.

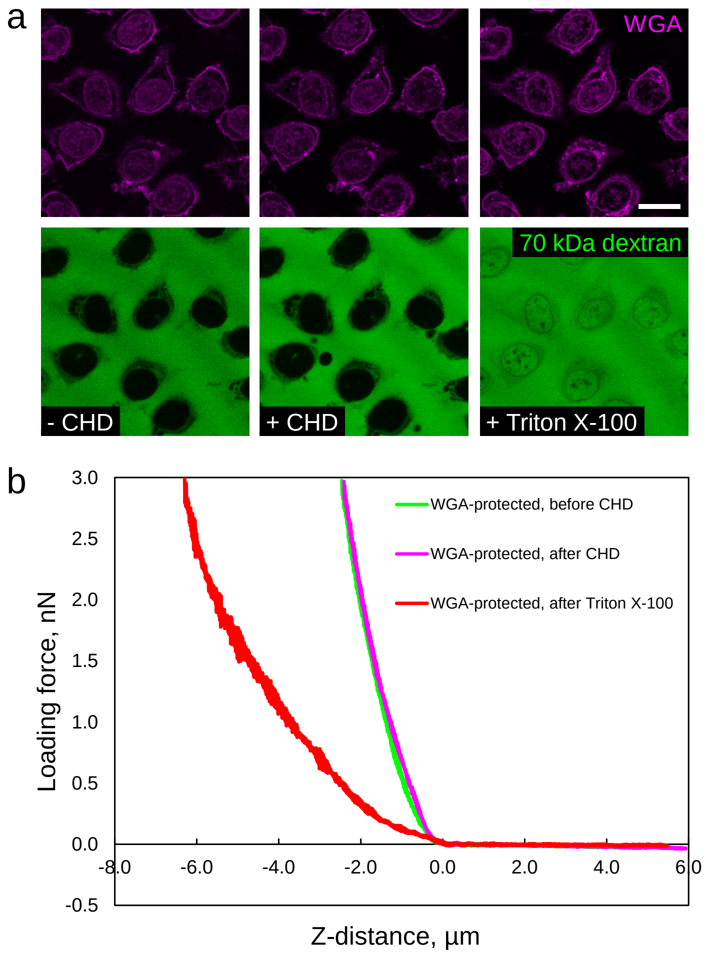

Figure S3. WGA affects the nuclear mechanics by protecting the NPC from the CHD activity but not through additional mechanical reinforcement. a) Visualization of WGA binding to N-acetylglucosamine residues in Ea.hy 926 endothelial cells in absence (left) and presence (middle) of CHD as well as in presence of Triton X-100 (right). Neither CHD nor Triton X-100 reduces the amount of bound WGA. WGA is able to protect the nucleocytoplasmic permeability barrier from the activity of CHD acting on the NPCs (compare bottom left and bottom middle). Conversely, WGA is unable to prevent Triton X-100 from attacking the lipid component of the nuclear envelope and compromising the permeability barrier (bottom right). b) Representative force distance curves from probing experiments on WGA-protected nuclei show that the intact barrier regardless of the presence or absence of CHD ensures similar levels of deformation (green and magenta curves). But as soon as the barrier is compromised by addition of Triton X-100 the deformation increases nearly 3-fold (red curve). The curves are taken from the measurements on one and the same cell at all three experimental conditions. Scale bar = 20 µm.

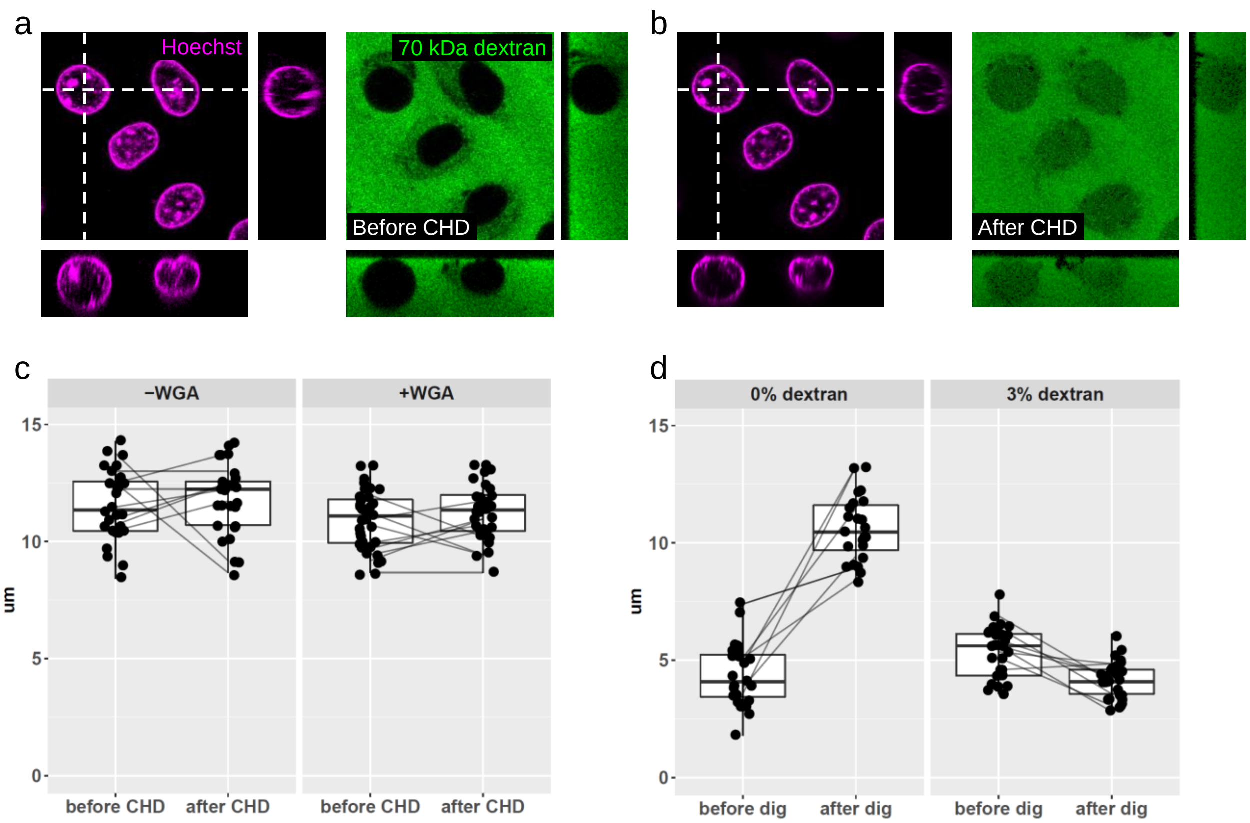

Figure S4. Only the molecules above the cut-off value of the nuclear envelope permeability barrier are able to exercise significant colloid-osmotic pressure and cause volume changes of the nuclei. a, b) Simultaneous staining of the chromatin and the surrounding medium demonstrates that neither the chromatin structure nor the nuclear geometry undergo significant changes upon addition of CHD. c, d) Statistical analysis supporting the notion that only the high molecular weight colloids are able to modulate the extent of colloid-osmotic pressure across the nuclear envelope. c) Addition of CHD causes no significant changes of the nuclear height whereas colloid-osmotic compensation with 3% dextran is able to restrict the fluctuations in nuclear geometry.
